# Gastric adenocarcinoma of fundic gland (chief cell predominant type) coexisting with well differentiated intestinal adenocarcinoma: A case report

**DOI:** 10.1097/MD.0000000000025861

**Published:** 2021-05-28

**Authors:** Lifeng Liu, Lin Han, Qingzhu Ma, Jinliang Zhang

**Affiliations:** a Department of Gastroenterology; b Department of Pathology; c Department of Hepatobiliary Surgery, Liaocheng Hospital, China.

**Keywords:** chief cell, gastric adenocarcinoma of fundic gland (chief cell predominant type), mucin-6, pepsinogen-I, tubular adenocarcinoma

## Abstract

**Rationale:**

Gastric adenocarcinoma of fundic gland (chief cell predominant type) (GA-FG-CCP) is a new, rare variant of gastric adenocarcinoma, which is characterized by mild nuclear atypia and specific immunohistochemical markers.

**Patient concerns:**

An 84-year-old Chinese man was referred to our hospital for endoscopic resection of a gastric lesion.

**Interventions:**

We performed endoscopic submucosal dissection, and successfully removed the lesion.

**Diagnosis:**

Esophago gastroduodenoscopy showed a slightly elevated lesion with a diameter of 22 mm in the posterior wall of cardia. Magnifying endoscopy with narrow band imaging revealed an abnormal microsurface and microvessels on the tumor surface. Endoscopic ultrasonography revealed a hypoechoic mass located in the first layer. The pathological diagnosis of the biopsy specimens indicated that the tumor was high grade intraepithelial neoplasia. The pathological diagnosis differed between the superficial and deeper part of the lesion. The superficial part was composed of a tubular structure with prominent atypia and was diagnosed as well differentiated intestinal adenocarcinoma. The deeper part was composed of a well-differentiated tubular adenocarcinoma mimicking the fundic gland cells, mainly the chief cells. The tumor cells showed mild nuclear atypia and was positive for pepsinogen-I (PG-I) and mucin-6 (MUC6). This deeper part was diagnosed as GA-FG-CCP.

**Outcomes:**

The tumor was successfully removed. This patient had no discomfort during the follow-up period (10 months).

**Lessons:**

We present a rare case of GA-FG-CCP coexisted with well-differentiated tubular adenocarcinoma. GA-FG-CCP exists in the deep mucosal layer and the muscularis mucosa, which could not be found under endoscopy, but could be discerned in pathology with mild nuclear atypia and special biomarkers.

## 1 Introduction

Gastric adenocarcinoma of the fundic gland type (GA-FG) is a rare variant of gastric adenocarcinoma. Tsukamoto et al^[1]^ in 2007 reported the first case of gastric adenocarcinoma with differentiation into chief cells within the fundic gland. Ueyama et al^[2]^ in 2010 proposed the concept of GA-FG. GA-FG is thought to develop in the upper or middle third portion of the stomach, arising from the gastric fundic gland region.^[3]^ Most of the GA-FG is solitary, with a diameter less than 1 cm.^[4]^ Most reports of GA-FG were from Japan and Korea, with only a few from China.^[5]^ Here, we report a case of GA-FG (chief cell predominant type) (GA-FG-CCP) in a Han Chinese colliding with a well differentiated intestinal adenocarcinoma.

## 2 Case report

### 2.1 Clinical and endoscopic findings

The study was approved by the local research ethics committee at the Liaocheng Hospital according to the principle of the Helsinki Declaration II. Written informed consent from this participant was obtained.

**Figure 1:**
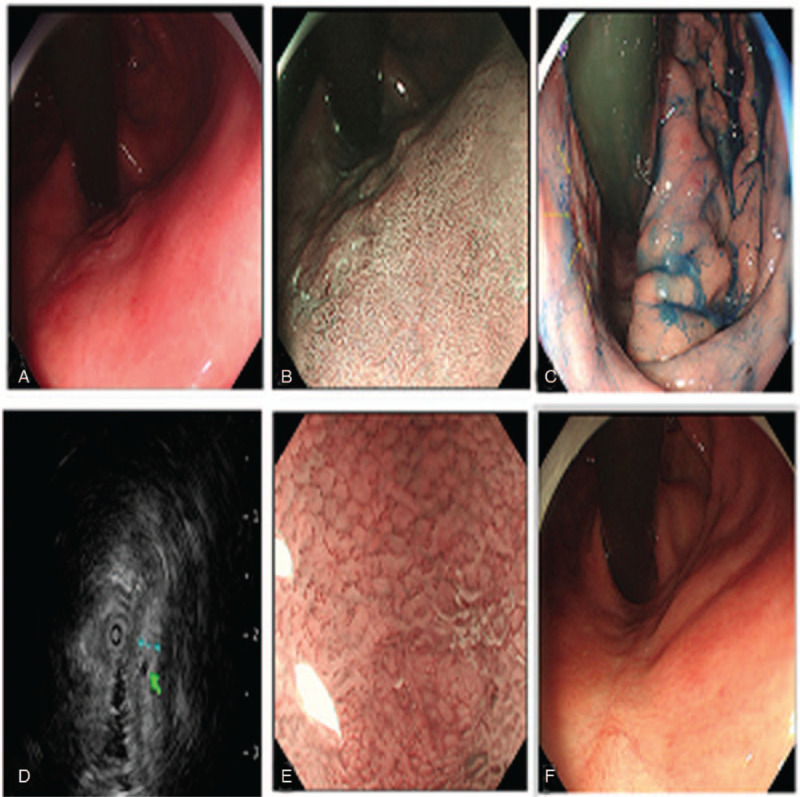
Endoscopic imaging of the lesion in the posterior wall of cardia. White light imaging showed a slightly elevated lesion in the posterior wall of cardia (A). NBI and indigo carmine spraying revealed a well demarcated line around lesion (B and C). EUS revealed a hypoechoic mass located in the first layer and no echo tube structures in the second layer (D). ME-NBI revealed an absent microsurface pattern and a fine network of irregular microvessels on the tumor surface (E). Endoscopic examination 3 months after ESD reviewed a scar (F). ESD = endoscopic submucosal dissection, EUS = endoscopic ultrasonography, ME-NBI = magnifying endoscopy with narrow band imaging, NBI = narrow band imaging.

An 84-year-old Chinese man visited a nearby hospital complaining of acid reflux. Esophago gastroduodenoscopy revealed an elevated lesion in the posterior wall of cardia, and histological examination of the biopsy specimens showed high grade intraepithelial neoplasia. He was referred to our hospital for further examination. Esophago gastroduodenoscopy showed a slightly elevated lesion with a diameter of 22 mm in the posterior wall of cardia, (Fig. 1A). The tumor was classified as type IIa according to the Paris classification. Narrow band imaging and indigo carmine spraying revealed a clear boundary around the lesion (Fig. 1B and 1C). Magnifying endoscopy with narrow band imaging revealed a partially absent microsurface pattern and a fine network of irregular microvessels on the tumor surface (Fig. 1E). Endoscopic ultrasonography revealed a hypoechoic mass located in the first layer and no echo tube structures in the second layer (Fig. 1D). Blood test results and imaging examination, including tumor markers and abdomen CT, were normal. No tumor metastasis was observed. Based on these findings, we believed the tumor to be an intramucosal gastric adenocarcinoma. Endoscopic submucosal dissection was performed, and the tumor was successfully removed. Delayed bleeding or perforation was not appeared after endoscopic submucosal dissection. Three months later, an endoscopic examination reviewed a scar and no sign of recurrence (Fig. 1F). This patient was alive and had no discomfort during the follow-up period (10 months).

### 2.2 Histological findings and cell differentiation markers

Immunohistochemistry was carried out for pepsinogen-I (PG-I) (a marker for chief cells), mucin-6 (MUC6) (mucous neck cell, pyloric glands), mucin-5AC (MUC5AC) (gastric foveolar cell), mucin-2 (MUC2) (goblet cell, a marker of intestinal phenotype), p53, and ki-67.

**Figure 2:**
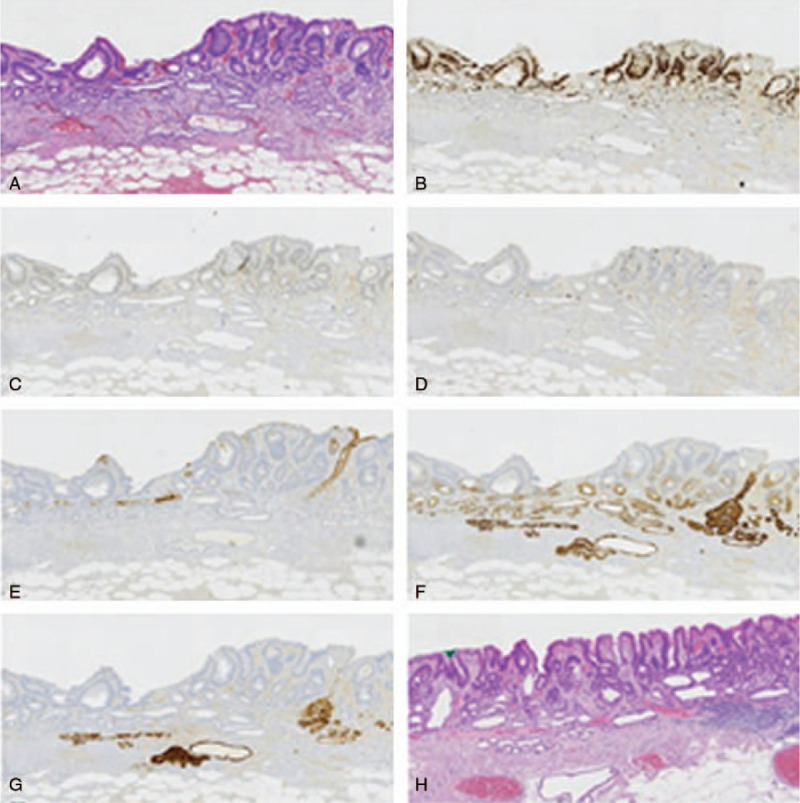
Histopathological and immunohistochemical findings. The superfical part was composed of a tubular structure with prominent atypia, and the nucleus is enlarged and elongated (A), positive for MUC2 (D), ki67 (B) and p53 (C), and negative for Muc5AC (E). The deeper part was composed of a well-differentiated tumor cells mimicking fundic gland cells, mainly the chief cells (A). The lesion was scattered, positive for MUC6 (F) and PG-I (G). MUC2 = mucin-2.

The histological findings differed between the superficial and deeper part of the lesion. The superficial part was composed of a tubular structure with prominent atypia, and the nucleus is markedly enlarged and elongated (Fig. 2A). This superficial part was diagnosed as well differentiated intestinal adenocarcinoma. Intestinal metaplasia and atrophy were observed at the mucosa surrounding the carcinoma. In immunohistochemistry, the tumor cells expressed scattered MUC2 (10%) (Fig. 2D), focally P53 (30%) (Fig. 2C), and highly Ki-67 (60%) (Fig. 2B). The tumor cells were negative for MUC5AC (Fig. 2E) and PG-I (Fig. 2G). The well differentiated intestinal adenocarcinoma was restricted within the lamina propria mucosae. In deeper part of the lesion, a well-differentiated tubular adenocarcinoma mimicking the fundic gland cells, mainly the chief cells, proliferated at the deep layer of the lamina propria mucosae and in muscularis mucosae (Fig. 2A). The tumor cells had slightly enlarged nuclei and showed mild nuclear atypia. Cystic gastritis was extensively observed at the deep mucosa and in muscularis mucosae (Fig. 2H). In immunohistochemistry, the deeper part cells were positive for MUC6 (Fig. 2F) and PG-I (Fig. 2G), negative for MUC2 (Fig. 2D) and MUC5AC (Fig. 2E). The Ki-67 labeling index score was extremely low. And the P53 is significantly lower than the well differentiated intestinal adenocarcinoma. This deeper part was diagnosed as GA-FG-CCP. The lesion showed no lymphatic or venous invasion.

## 3 Discussion

GA-FG is composed of well-differentiated columnar cells with pale basophilic cytoplasm and mild nuclear atypia that mimic the fundic glands. MUC6 and pepsinogen I are strongly expressed, and typical GA-FG is negative for CD10 and MUC2. However, some reports have demonstrated that GA-FG was shown to have submucosal invasion. The cases with follow-up data, 12-year and 19-year natural history of GA-FG was observed respectively, and no morphological changes were observed.^[6,7]^ GA-FG is considered to have a low-grade malignancy. In contrast, in some cases, high-degree atypia and high-grade malignancy has been reported, and it was speculated that GA-FG that differentiated to several directions had a higher malignant potential. GA-FG can coexist with other types of gastric adenocarcinoma. Keitaro Takahashi et al^[8]^ in 2017 reported the first case of GA-FG colliding with well differentiated adenocarcinoma. Keita Kai et al in 2018 report a case of gastric adenocarcinoma of fundic gland type with signet ring cell carcinoma component.^[9]^

The distinctive feature of our case was the coexistence of the well-differentiated tubular adenocarcinoma and GA-FG-CCP. The superficial part was well-differentiated tubular adenocarcinoma, expressed the intestinal type of MUC2. Atrophy and intestinal metaplasia were also observed in the background mucosa. The above suggested that this part originated from the intestinal metaplasia, and had an intestinal phenotype. The partially absent microsurface and the fine network of irregular microvessels correspond to the well-differentiated tubular adenocarcinoma in histopathology. The deeper part was composed of a well-differentiated tumor cells mimicking fundic gland cells, mainly the chief cells. The nuclear atypia was mild and the lesion was scattered, mixed with cystic gastritis. It is hard to distinguish. Immunohistochemistry can help to identify. In immunohistochemistry, this part was positive for MUC6 and pepsinogen-I. MUC6 and pepsinogen-I were special biomarkers of fundic gland cells. Therefore, the deeper part was referred to as GA-FG-CCP. There is no clear boundary between the well differentiated adenocarcinoma and the GA-FG. This is unusual finding for GA-FG. The growth pattern of our case is significantly different from that reported by Keitaro Takahashi et al^[8]^ The lesion reported by Keitaro Takahashi was 2 different components (0-I + 0-IIa). The 0-I lesion was referred as GA-FG, and the 0-IIa was referred as well differentiated adenocarcinoma. There is a clear boundary both in endoscopy and histology between 0-I and 0-IIa lesion. GA-FG existed in the deep mucosal layer and the muscularis mucosa, and it is difficult to identify under endoscopy. For the pathological features, GA-FG with mild nuclear atypia, especially existing simultaneously with deep cystic gastritis, is easily overlooked. The pathologist should pay attention to discern this lesion and judge the depth of tumor invasion based on the depth of GA-FG. GA-FG is a well-differentiated neoplasm and has a good prognosis, while the etiopathogenesis is unclear. Coexistence of the 2 lesions is rare, only a few information on the early stage is available to date.^[8,9]^ To understand the co-existing lesion, similar cases should be studied and long term follow-up is necessary in the future.

## Author contributions

**Conceptualization:** Jinliang Zhang.

**Data curation:** Lifeng Liu, Lin Han.

**Formal analysis:** Jinliang Zhang.

**Investigation:** Lifeng Liu, Lin Han.

**Methodology:** Lifeng Liu, Lin Han.

**Supervision:** Qingzhu Ma.

**Validation:** Lin Han.

**Writing – original draft:** Lifeng Liu, Lin Han.

**Writing – review & editing:** Lifeng Liu, Lin Han, Qingzhu Ma, Jinliang Zhang.
